# Electronic cigarettes as a harm reduction strategy among patients with COPD: protocol for an open-label two arm randomized controlled pilot trial

**DOI:** 10.1186/s13722-021-00284-0

**Published:** 2022-01-06

**Authors:** Elizabeth R. Stevens, Lei Lei, Charles M. Cleland, Mahathi Vojjala, Omar El-Shahawy, Kenneth I. Berger, Thomas R. Kirchner, Scott E. Sherman

**Affiliations:** 1 New York University Langone Health, New York, NY USA; 2 New York University School of Global Public Health, New York, USA

## Abstract

**Background:**

Smoking cessation is the most effective means of slowing the decline of lung function associated with chronic obstructive pulmonary disease (COPD). While effective smoking cessation treatments are available, they are underutilized and nearly half of people with COPD continue to smoke. By addressing both nicotine and behavioral dependence, electronic cigarettes (EC) could help people with COPD reduce the harm of combustible cigarettes (CC) through reductions in number of Cigarettes per Day (CPD) or quitting CC completely. The purpose of this pilot study is to identify barriers and facilitators to the use of and assess the preliminary effectiveness of EC as a harm reduction strategy among people with COPD.

**Methods:**

In an open-label two-arm randomized controlled trial pilot study, 60 patients identified as smokers with a COPD diagnosis via electronic health records from a large urban health center are randomized in a 1:1 ratio to either standard care [counseling + nicotine replacement therapy (NRT)] or counseling + EC. The NRT arm will receive nicotine patches and nicotine lozenges for 12 weeks. The EC arm will receive EC for 12 weeks. Both cohorts will receive counseling from a licensed mental health counselor. Using ecological momentary assessment, participants will report their use of CC in both arms and EC use in the EC arm daily via text message. Primary outcomes will be feasibility and acceptability of intervention, and secondary outcomes will be reduction in CPD and change in COPD symptoms as measured by COPD Assessment Tool (CAT) score at 12-weeks. EC displacement of CC. To explore attitudes towards the use of EC as a harm-reduction strategy for patients with COPD, interviews will be performed with a sample of participants from both study arms.

**Discussion:**

Despite decades of availability of smoking cessation medications, nearly half of people with COPD still smoke. This study aims to address the unmet need for feasible and effective strategies for reducing CC use among those with COPD, which has the potential to significantly improve the health of people with COPD who smoke.

*Trial Registration* ClinicalTrials.gov Identifier: NCT04465318.

## Introduction

Despite considerable progress, smoking remains the leading preventable cause of death in the United States (US), causing 480,000 deaths and $300 billion in health-related economic losses each year [1]. In the US, chronic obstructive pulmonary disease (COPD) represents one fifth of all smoking-related deaths [2] and over 16 million people in the US have COPD [3]. Globally, the burden of COPD is even greater, with COPD projected to be the third largest cause of death by 2030 [4].

Among those with COPD who smoke, smoking cessation is the most effective means of slowing the decline of lung function and overall disease progression [5, 6]. Patients at all stages of COPD benefit from smoking cessation [6]. Although effective smoking cessation treatments are available, after an initial quit relapse is high [7] and nearly half of smokers with COPD are still smoking [8]. To encourage more patients with COPD to quit, alternate smoking cessation tools are needed.

By addressing both nicotine and behavioral dependence, electronic cigarettes (EC) may represent a more appealing alternative smoking cessation tool for many smokers [9–11], and when paired with behavioral therapy were shown to be nearly twice as effective in helping people quit smoking (18.0% vs. 9.9%) as other nicotine replacement therapies (NRT) [12]. While not all EC smoking cessation studies have shown higher quit rates, there is moderate‐certainty in the evidence that quit rates are higher in people randomized to nicotine EC than in those randomized to nicotine replacement therapy (NRT) [13]. Switching from combustible cigarettes (CC) to EC has been associated with improved lung function in patients with COPD [14, 15]. FDA-approved smoking cessation medications have been less effective for patients with COPD [16]. This may be because pharmacotherapy interventions do not replace the behavioral ritual associated with CC use, nor deliver nicotine as rapidly as CC [17, 18].

Switching to EC may be beneficial in people with COPD. Little is known about health consequences of EC use among smokers with COPD [19] Polosa and colleagues (2016) conducted a retrospective chart review of 48 patients with COPD who had reported regular daily use of ECs on at least two follow-up visits at 12-, 24-, 48-, and 60-months. Compared to regularly smoking COPD patients, switching from CC to EC was associated with improved lung function [14] and benefits persisted long-term [15, 20]. At 60-month follow-up, people who switched to EC had a significant decrease in COPD exacerbations (from 2.3 to 1.1) and in scores on the COPD assessment tool (from 21.5 to 17.5). People who switched to EC had significant and constant improvement in lung function compared to continued smokers at all follow-up time points.

EC are not risk free and are undeniably harmful to non-smokers [21, 22]. Nevertheless, evidence suggests the benefits of EC in helping with CC cessation and harm reduction substantially outweigh their potential harms [18, 23, 24]. A harm reduction approach seeking to achieve switching from CC to EC may be a more pragmatic approach than complete nicotine abstinence for those with high levels of addiction thus making EC use particularly appropriate for patients with COPD who continue to smoke [25]. Those with COPD could reduce CC harm by reducing the number of cigarettes per day (CPD) or completely switching to EC. The overarching goal of this study is to address the unmet need for feasible and efficacious strategies for reducing CC use among those with COPD, which has the potential to significantly improve the health of those with COPD who smoke.

This study aims to collect information on the feasibility and acceptability of an EC harm-reduction intervention among those with COPD who smoked. It also seeks to explore the preliminary effectiveness of the intervention on reduction of CPD and improved respiratory health.

## Methods

In conjunction with a qualitative study using in-depth interviews with 20 study participants, the proposed study seeks to conduct an open-label randomized controlled trial (RCT) comparing the effect of standard care (NRT + counseling) to EC + counseling on reduction of CPD in 60 smokers with COPD. Specifically, this study aims to: (1) determine the feasibility and acceptability of an EC intervention for CC harm reduction among a COPD population; (2) estimate EC-related reduction in CPD 12 weeks post baseline; (3) measure engagement with the text-messaging-based smoking diary; and (4) estimate the effect of EC on reductions in COPD symptoms. Aims examining intervention effect are exploratory as the study is not powered to detect significant differences in results. The study protocol has been approved by the NYU Langone Institutional Review Board (IRB).

The primary hypothesis to be tested is: EC is a feasible and acceptable harm-reduction intervention among patients with COPD who smoke. Secondary hypotheses include: (1) An EC harm-reduction intervention will be more effective than NRT in reducing CPD in patients with COPD who smoke; and (2) An EC harm-reduction intervention will be more effective than NRT in reducing COPD symptoms in patients with COPD who smoke.

### Participants

The sample will consist of 60 adults with COPD who smoke. These participants will be recruited from the electronic health record (EHR) of the New York University Langone Health system (NYULH), a private hospital system serving New York, New Jersey, and Connecticut with approximately 6.8 million active patients, including approximately 45,000 with a COPD diagnosis. The prevalence of current smoking among NYULH patients with a COPD diagnosis is 21%, which is about 50% higher than the prevalence of smoking in the general New York City population [26].

#### Inclusion criteria

Potential participants will be included if they (1) have an ambulatory ICD-10 code for COPD in the last 12 months; (2) a COPD Assessment Tool (CAT) [27] score ≥ 10; (3) are aged 21 to 75 years (the legal age for purchasing EC is 21); (4) are a current CC smoker (more than 5 packs in a lifetime; smokes 4 or more days/week); (5) smoke at least 5 CC per day on days they smoke CC; (6) are motivated to quit smoking (at least a 5 on a 10-point Contemplation Ladder [28]); and (7) possess a phone with text messaging capabilities.

#### Exclusion criteria

Potential participants will be excluded if they (1) have a CAT score ≥ 30 (representing severe COPD) [27] or < 10 (representing mild COPD); (2) report using NRT or EC within the last 14 days; (3) have a medical condition (e.g. unstable angina/heart disease) precluding use of nicotine patch or gum as determined by the study physician or by their treating physician; or (4) are pregnant (as determined by urine pregnancy test for women under age 52) or breastfeeding (self-reported). Women of childbearing age must also be willing to use an approved form of birth control during the course of the study if not practicing abstinence. Approved birth control methods include: hormonal birth control (e.g. “the pill”), barrier methods (e.g. condoms, diaphragm), and intrauterine devices (IUDs). Use of birth control will not be directly assessed, however, as required by the IRB, if a participant becomes pregnant during the course of their study participation they will be withdrawn from the study and referred to their personal physician for further smoking cessation advice.

#### Recruitment

Participants will be recruited from a list of CC-smokers with COPD in the NYULH EHR who have not opted out of being contacted for clinical research. Prior to contacting a patient, the treating physician will be notified of their patient’s potential participation in the study. Patients will not be contacted if their physician responds stating that their patient should not participate. Participation will be at the discretion of the treating physician, who will be encouraged, but not required to disclose the reason for exclusion. Potential reasons for exclusion are expected to include severe psychiatric illness or comorbidities where nicotine replacement would be contraindicated.

To reach out to potential participants we will use a multimodal strategy, first sending a mailing and following up with a phone call. Potential participants will receive a letter introducing the study along with the Informed Consent form. For potential participants who have an active MyChart account (an online system that allows patients to access their medical records and communicate with their physicians), we will also send a MyChart message describing the study. One week later, we will call them by telephone to complete the eligibility screening. We will make up to 5 call attempts to each potential participant. Call attempts will be made over a span of two weeks and be scheduled at varying days of the week and times of day. Eligible participants will be invited to give informed consent digitally via the REDCap (Research Electronic Data Capture) e-Consent Framework, a secure web application for building and managing online surveys and databases [29].

### Trial design

**Fig. 1:**
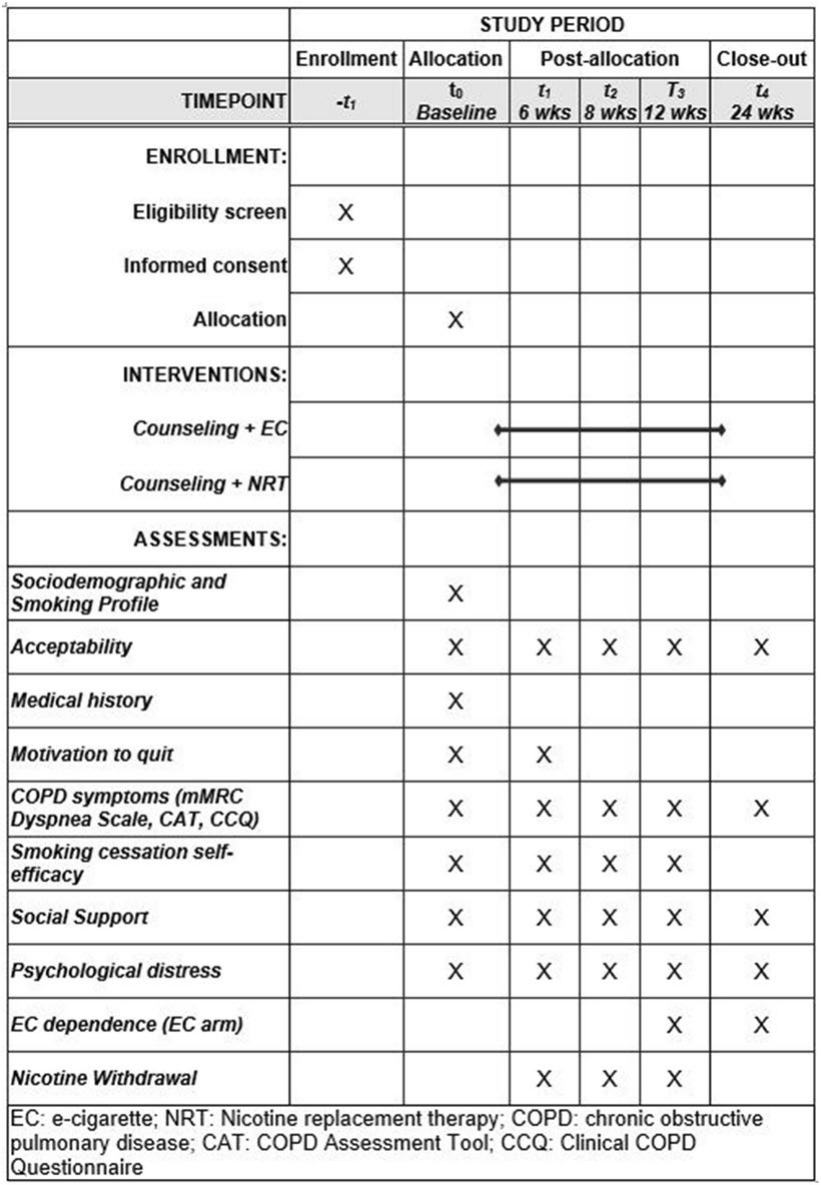
SPIRIT flow diagram: schedule of enrolment, interventions and assessments

After completing the baseline survey on tobacco use, physical health, and mental health, we will use a two-arm study design to randomize 60 patients to counseling + NRT (standard care) or counseling + EC. Participants will receive four counseling sessions in 12 weeks; each session will follow a study visit monitoring any physical or mental changes and side effects from using NRT or EC. All participants will complete a follow-up survey at 24 weeks (Fig. 1). During the 12 weeks of study participation, participants will report their tobacco use daily by responding to automated text messages serving as a smoking diary. We will also invite 20 participants for an in-depth interview on their study experience, outcome expectations, EC perceptions, and barriers to smoking cessation.

#### Outcomes

Our primary outcomes include: (1) feasibility of intervention, and (2) acceptability of intervention. Secondary outcomes include: (1) change in number of self-reported CC per day based on daily reports that are provided for 84 days post-baseline and (2) change in pulmonary symptoms based on CAT scores at 12 weeks.

#### Randomization

After eligibility screening, participants will be randomized into one of the two study arms (EC or NRT; ratio 1:1) using minimization methodology. This approach has been found to achieve significantly better covariate balance than other randomization methods with a small sample size [30]. Randomization will be stratified by three key variables CAT score (10–19 and ≥ 20), CPD (< 20 and ≥ 20), and sex and will occur using a REDCap based system which includes verification of eligibility criteria prior to the assignment of a study ID and intervention group. Due to the nature of the study, participants are not blind to their randomization allocation.

#### Intervention

##### Intervention arm: EC + counseling

At baseline, intervention arm participants will be provided with a kit containing approximately one month’s supply of EC. If the initial supply does not cover the full month’s use, participants will be able to request an additional supply or change of flavors. A new supply of EC will be distributed regularly during each study visit and as needed for 12 weeks. Participants will be asked to provide regular text messaging check-in reports 4 times over the course of each day to report CC and EC use.

###### Electronic cigarettes

We will use the NJOY “Daily”, an electronic nicotine delivery system that heats an e­liquid to yield an inhalable aerosol. It is manufactured by NJOY LLC, the only major EC company not owned wholly or in part by a tobacco company. NJOY also manufactures the NIDA Standardized Research E-Cigarette [31]. The product is a daily use (approx. 350 puffs) disposable EC with 4.5% nicotine by weight intended to be discarded after its e­liquid has been depleted. One NJOY Daily has the same nicotine content as 1.5 packs of CC. The NJOY Daily is a factory-sealed closed system, which prevents tampering with its designated e-liquid. It is available in two flavors (tobacco and menthol). Participants can choose between tobacco flavor, menthol flavor, or both based on their preference. EC products are all regulated as consumer tobacco products by FDA’s Center for Tobacco Products (CTP). This study examines EC as a harm-reduction strategy, not a smoking cession device. Therefore, the FDA does not require an investigational device exemption (IDE).

##### Control arm: NRT + counseling

Participants in the control arm will be provided with a kit containing a month’s supply of NRT. If the initial supply does not cover the full month’s use, participants will be able to request an additional supply. A new supply of NRT distributed regularly and as needed for 12 weeks. Participants will be asked to provide regular, and very brief, text message check-in reports over the course of each day to report CC and NRT use.

###### Nicotine replacement therapy

Participants with CPD ≥ 10 will be given 21 mg nicotine patches and those with CPD < 10 will be given 14 mg or 7 mg nicotine patches. All participants in this group will also be given 4 mg nicotine lozenges. We will provide sufficient NRT products to participants for 12 weeks.

##### Behavioral counseling

At baseline, all participants will receive a 30-min counseling session delivered by telephone from a counselor trained in motivational interviewing and smoking cessation treatment tailored to the participant’s designated EC or NRT arm. The proposed counseling program has been shown to be acceptable and effective for achieving abstinence in a previous smoking cessation program [57]. Counseling sessions will be similar between the two arms and use the same counseling techniques. Counseling will be provided by a licensed mental health counselor with extensive experience in the field of health psychology and behavioral health research. The same counselor will contact each participant by telephone at 2 weeks and 1 and 2 months to deliver additional 10–15 min counseling sessions, for a total of 4 counseling sessions. For this pilot, a single counselor is expected to complete sessions for all participants, however if additional counselors are used they will be trained to ensure consistent intervention delivery and will evenly split participants between arms.

The purpose of the counseling is to: (1) discuss the impact of continued smoking on their COPD; (2) deliver motivational enhancement for switching from CC to EC (EC arm) or smoking cessation (NRT arm); (3) promote self-efficacy; and (4) inform participants about EC and NRT use. The counseling protocol will be characterized by (1) Motivational enhancement – We will include motivational enhancement in the initial counseling and each of the telephone calls to increase patient motivation to switch from CC to EC or quit smoking; (2) Problem-solving therapy—this approach—endorsed by the national smoking cessation guidelines [32]—will be adapted to focus on helping the smoker identify and solve challenges in switching from CC to EC or quitting smoking.

The counselor will review each participant’s smoking patterns and offer tailored behavioral and environmental change strategies, including specific options such as substituting EC for CC at work, in the home, or least favorite or most favorite CC of the day. Participants will be encouraged to carry their EC at all times for use when avoiding smoking triggers, for use as oral or manual replacement, and provided with other strategies to manage urges. Since there is inadequate evidence that any one of these strategies was more effective than another, participants will be encouraged to choose those that are most appealing. Goal setting and barrier identification will be used to help participants improve confidence in their ability to completely switch to exclusive EC use. Participants will also be provided with information regarding the health risks associated with smoking and advice on smoking cessation will be freely available to all participants at all visits.

Since choosing to use treatment and ultimately to quit is affected by personal, social (e.g., social support) and cognitive (e.g., self-efficacy) factors, counseling will be guided by the framework of Social Cognitive Theory (SCT). SCT has been useful as a theoretical model for understanding barriers and facilitators predicting CC harm reduction [34], particularly with vulnerable groups [35, 36]. Data collected in the in-depth interviews will enable us to refine our counseling content to address factors that may influence CC harm reduction among COPD CC smokers, including social environmental influences, self-efficacy, and outcome expectations [33], as well as other issues specific to specific to COPD.

#### Study visits

Participants will complete study visits by telephone at baseline, 2-, 4-, 6-, 8-, 12- and 24-weeks. At each visit, research assistants will complete a checklist for all activities including completing surveys, assessing for potential harm, monitoring use of non-study supplied nicotine containing products, and confirming adequate supply of EC/NRT.

##### Participation incentives

Participants will have the potential to receive a total of $120 for participating in the study. Participants will not be compensated for study visits or counseling sessions. However, coinciding with study visits, participants will receive $10 for completion of a survey at baseline, 6-, 8-, and 12-weeks, and $20 for completion of a survey at 24-weeks. Additionally, participants can receive up to $40 for completing their daily smoking diaries via text messages ($20 if at least 60% of assessments are completed, $40 if at least 80% are completed). Finally, participants who complete an in-depth interview will receive $20.

##### Data collection and measures

Measures will be collected via survey at baseline and at 6-, 8-, 12-, and 24-weeks, and on a daily basis via the ecological momentary assessment (EMA) text messaging program through week 12. At baseline, all subjects will be surveyed to assess demographics, social support [37], smoking history and habits, smoking cessation self-efficacy [38], tobacco dependence [39], and the treatment offered and used in the prior 12 months, as well as their attitudes toward EC and types of smoking cessation treatment (medications, counseling, and texting). To maximize compatibility with other data and increase the potential for combination with other samples, when possible measures are drawn from NIH initiatives for measure standardization, including the PhenX Toolkit [40], the NIH Toolbox [41], and the Patient Reported Outcomes Measurement Information Systems Initiative (PROMIS) [42]. Relevant tobacco-related measures are drawn from the Population Assessment of Tobacco and Health (PATH) study [43]. Measures not included in the NIH initiatives, such as COPD severity (CAT score), past treatment, and general medical history were derived from the literature. All data will be managed in RedCap.

##### Feasibility and acceptability

The feasibility and acceptability of an EC harm-reduction intervention for patients with COPD who smoke will be assessed with five measures: (1) eligibility and acceptance rate (number accepting enrolment/number eligible); (2) proportion of participants engaging in follow-up visits; (3) retention rate at the end of 12-week treatment period and 24-week follow-up (retained/n); (4) dose of intervention received (e.g., number of counseling sessions); and (5) acceptability of the intervention (satisfaction with EC/NRT) as measured on a 5-point Likert scale. EMA response rate will also be measured.

##### Tobacco product use

CC and EC/NRT product use will be measured as part of a text messaging EMA protocol serving as a smoking diary that incorporates a behavioral “coverage” strategy, designed to maximize the odds that all behavioral events of interest are documented accurately and minimize missing data [44]. Daily recording of product use via EMA allows for measurement of dual use of products and observation of switching patterns. This also provides a measure of CPD over time. After the baseline assessment, participants will be asked to record daily nicotine use by responding to text message prompts. The coverage design prompts participants to provide regular, and very brief, check-in reports via text message over the course of each day, wherein they are asked to report use of EC or NRT based on their study arm and CC over the window of time elapsed since their last report (~ 4 h). One item measures of CC craving and EC/CC satisfaction are collected at each report.

**Fig. 2:**
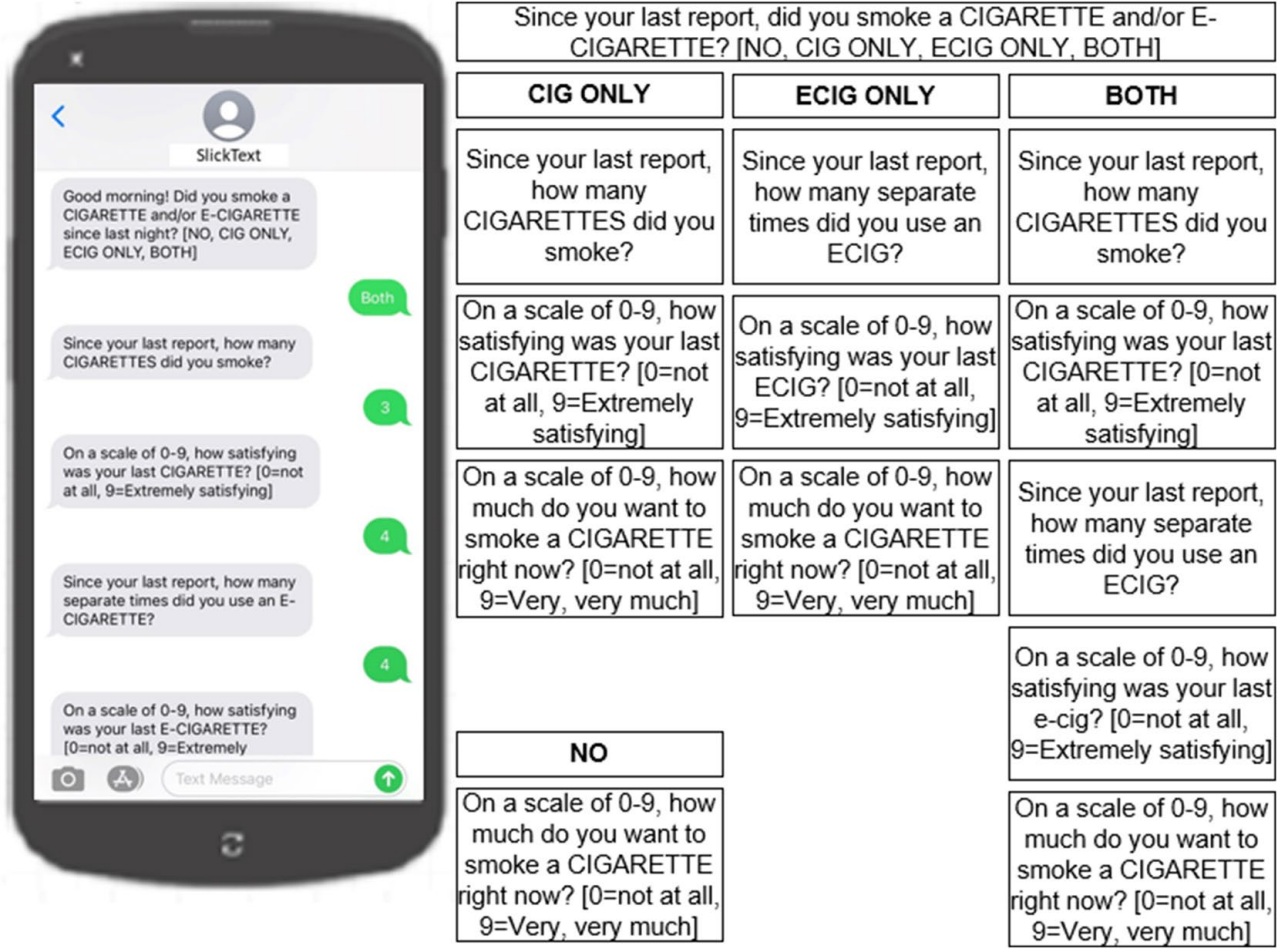
Daily text message script

Combined in succession, these coverage reports produce near-complete and consistent use reports over relatively extended periods, partly because they require only seconds for subjects to complete. Text message smoking diary prompts will be automated using SlickText Workflows software and their associated text distribution platform. Frequency of text message prompts will be four per day, corresponding to time windows breaking the day into “Early/Lunch/Afternoon/Evening” periods. There is minimal burden since each data collection instance takes an average of one minute to complete each session. Participants will receive a text message prompt asking them to complete an assessment (Fig. 2). The system is programmed to prevent skipping items or entering out-of-range values. Data is continuously synchronized with a secure web-based HIPAA compliant server. All entries will be time- and date-stamped. The system will record the time it took the participant to respond (response delay), time to completion, and missed prompts. All participants will be instructed how to document their daily tobacco use.

##### COPD symptoms

COPD symptoms, including dyspnea and functional status, will be measured by self-report using three tools including: mMRC (Modified Medical Research Council) Dyspnea Scale examining breathlessness walking [45], COPD Assessment Tool (CAT) score measuring symptoms such as coughing chest tightness, and energy levels [27], and the Clinical COPD Questionnaire (CCQ) measures health status and can be used to assess health-related quality of life [46].

#### In-depth interviews

We will conduct in-depth interviews lasting approximately 30 min with 10 participants in each arm (n = 20) after study visit six (12 weeks post-baseline). Participants will be selected as a convenience sample. At the time of the 6th study visit participants will be invited to participate in an in-depth interview, which will be scheduled and performed via telephone. Recruitment will continue until an n = 10 for each arm is achieved. Excluding specific treatment product satisfaction (i.e. NRT vs EC) prompts, interview questions will be identical for both arms. These assessments will help to gain a more in depth understanding of the participant’s experience, intervention satisfaction, attitudes towards EC, and intentions to quit. These interviews will provide additional insights about the barriers and facilitators of EC use among people with COPD, and how we may refine our approach to enhance retention and outcomes. The interviews will supplement the quantitative data to provide a more in-depth understanding of the feasibility and acceptability of the intervention. Interviews will cover topics such as aspects they like/dislike; features of the intervention that should be modified; their experiences using EC/NRT; intentions of using EC after the intervention (NRT regimen is typically 2–3 months); and whether their COPD symptoms interfered with their ability to engage in the intervention. The interviews will also be used to further adapt the behavioral counseling manual to an EC intervention for a future trial. The interviews will be audio recorded and participants will be required to provide audio consent prior to conducting the interview. Recordings will be transcribed with personal identifiers removed, and the actual recording will be destroyed at the end of the study.

#### Statistical methods

Before addressing the study aims, data will be summarized numerically using descriptive statistics. Comparison of the baseline characteristics between the study arms will be performed using chi-square tests for categorical data and t-tests or nonparametric tests for ordinal or continuous data. Preliminary analyses will investigate possible differences on the primary outcomes by gender and race/ethnicity. If differences appear, race/ethnicity and/or gender will be entered as covariates. Exploratory analyses will be conducted where appropriate to better understand the findings. Otherwise, analyses will collapse across race/ethnicity and gender. All data will be presented in aggregate and de-identified prior to publication.

Feasibility and acceptability will be summarized using descriptive statistics. Simple logistic regressions, including ordinal and multinomial models, will be used to compare the characteristics of participants reporting high and low acceptability. We will evaluate the impact of EC vs. NRT on the reduction in self-reported CPD and CAT Score, at 12 and 24 week follow-up assessments, with and without the addition of covariates. CPD at baseline and 12 weeks will be extracted from EMA data, while the 24-week follow-up will be self-report from the telephone survey. The impact of EC vs. NRT on change in CPD and CAT Score will be estimated using a Poisson or negative binomial regression model [47] with follow-up CPD/CAT Score regressed on baseline CPD/CAT Score and an indicator variable of study condition (EC vs. NRT).

This study is not meant to provide a definitive test of intervention efficacy and will be used to inform future sample sizes for a larger trial [48, 49]. In the primary analysis the sample size is anticipated to provide 80% power to detect a difference of 0.73 points in intervention satisfaction between study arms. For other primary measures, the sample size is anticipated to provide exploratory results to examine observed trends, but not statistically significant difference. For secondary analyses of effectiveness, in a Poisson generalized linear model for the number of CC reduced from baseline to follow-up, we will have greater than 90% power to detect a rate ratio of RR = 1.60 (e.g., a reduction by 5 CC per day in the NRT condition vs. 8 in the EC condition). If there is marked overdispersion of the number of CC per day reduced (κ = 0.2), we will still have greater than 80% power to detect RR = 1.80 (e.g., 5 vs. 9 CC per day reduction). Similarly, without considering covariates, we have 80% power to detect a difference of at least 2.7 on the CAT score, and with effective covariates, we have 80% power to detect a difference of 2.2. Translated to standardized mean differences, these are modest effects (d = 0.33 and d = 0.26). In secondary analyses, we will consider CPD collected daily over a 14-day period prior to the end of the 12-week follow-up. This will increase the power by increasing sample size providing for greater than 90% power to detect a rate ratio of RR = 1.20 (e.g., a reduction by 5 CC per day in the NRT condition vs. 6 in the EC condition). If there is marked overdispersion of the number of CC per day reduced (κ = 0.2), we will still have greater than 80% power to detect RR = 1.40 (e.g., 5 vs. 7 CC per day reduction).

##### Qualitative data analysis

Data collected from interviews will be recorded and transcribed for analysis. Data will be coded according to the principles of the framework for applied policy research [50]. Directed content analysis technique with deductive coding will be used [51]. The developed codes will represent each of the relevant constructs with additional codes developed from themes arising from the data, using the principles of grounded theory [52]. Interview transcripts will be analyzed with Atlas.ti 9.0 for thematic analysis [53]. Inter-coder reliability will be calculated using the first two transcripts. Disagreements will be discussed and additional transcripts will be reviewed until 90% inter-coder reliability is achieved. When coding is complete, the outputs of quotations with each code will be examined and summarized into themes. A sample of 20 interviews was determined a priori to potentially achieve theme saturation [54].

#### Harm

EC are not deemed risk free, with some studies suggesting adverse pulmonary effects [21, 22], and some brands of the liquid nicotine contain toxicants and carcinogens found in CC [55–57]. To address these concerns any minor or major events associated with the intervention or NRT arms will be screened for through survey and open-ended questions. At each study visit, participants will be asked about any changes in their medical status, potential side effects of NRT/EC use, psychological distress [58], nicotine withdrawal [59], and in the intervention arm EC dependence will be assessed [60]. Any adverse events or unintended effects detected will be reviewed by a researcher and a study physician.

## Discussion

Among those with COPD who smoke, the most effective means of slowing COPD progression is smoking cessation [5, 6]. In spite of decades of progress in tobacco control, nearly half of people with COPD still smoke CC [8]. The overarching aim of this study is to address the unmet need for feasible and effective strategies for reducing CC use among those with COPD, which has the potential to significantly improve the health of those with COPD who smoke. EC could provide an additional tool for harm reduction in adult smokers who have greater difficulty quitting. This research has implications for both the clinical treatment of COPD as well as public health tobacco use treatment policy. The data from this pilot study will provide the foundational knowledge necessary to determine the feasibility of an EC harm-reduction intervention in this population and inform modifications to intervention counseling tools to address the specific needs of this population. If found to be feasible and acceptable, a fully powered trial is anticipated and the counseling program will be manualized.

EC may lead to more use than traditional NRT. Despite 30–40 years of availability, smokers often have very limited enthusiasm for NRT, and decades of interventions have not had a large impact on that. Claims that EC are a harm-reducing alternative to smoking resonate among current smokers [61–64]. In addition, smokers often prefer the experience of using EC compared to CC [65], and there are indications that many smokers find EC more appealing than other smoking cessation aids (such as NRT) [9–11]. This preference indicates the population reach and impact of EC could prove greater than traditional pharmacotherapies.

The relative harm of EC is significantly less than CC, and the public health implications of adult smokers switching to EC are great. While not without some health risks [66–68], all available evidence indicates that EC are safer than CC. There is strong evidence suggesting that the benefits of EC helping with CC cessation and harm reduction substantially outweigh their potential harm [18, 23, 24]. Unlike CC, EC are not associated with coronary heart disease or myocardial infarction [69]. Replacing most CC use with EC use in the US could result in 1.6–6.6 million fewer premature deaths and 20.8–86.7 million fewer life-years lost over a 10-year period [24]. While EC uptake by adolescents is of great concern, this modelling study found that the gains in adults outweighed the harm in adolescents by a large margin in all sensitivity analyses. Warner and Mendez similarly found that over a wide range of plausible parameters, “potential life-years gained as a result of vaping-induced smoking cessation are projected to exceed potential life-years lost due to vaping-induced smoking initiation” [18]. However, few studies have looked at the extent to which smokers will substitute EC for CC, and these studies have been small [70, 71].

A harm reduction approach with the goal of achieving CC switching may be a more pragmatic approach, making EC use particularly appropriate with COPD [25]. EC represent a potentially effective harm reduction tool that is safer than smoking CC [18, 23, 24]. Smokers with COPD, however, tend to be older and may have a higher level of addiction to nicotine than the average smoker and the feasibility and preliminary effectiveness of an EC harm-reduction strategy in a COPD population has not been explored. More research is needed to evaluate the role of EC in the smoking patterns of adults and the impact of switching from CC to EC in people with COPD. This pilot study will serve to fill these knowledge gaps and provide the groundwork for future research in this area.

Our study protocol has a few limitations. First, as a pilot study the protocol is not powered to detect small differences in CPD or CAT Scores between the NRT and EC arms. Second, CAT Score is not the gold standard for the assessment of respiratory health. If this study is successful, future research needs to include assessment of airflow by spirometry, as well as by assessment of small airway function using respiratory oscillometry. While FEV1 change is not typically observable in early stage COPD patients, oscillometry may identify the presence of small airway injury in this population [72]. Adherence to study medications is not directly measured, therefore analyses are limited to intent-to-treat rather than per-protocol. The NJOY EC used in the study is not representative of all EC available and additional research using alternate products is called for. EC contain nicotine, which is an addictive substance and therefore there is potential for participants to continue EC use after the study is complete. The counseling protocol does not include EC cessation. Future research on EC cessation methods and continued EC use after switching is called for. Finally, all study measures are self-report. Future studies should include bio-confirmation of CC reduction such as CO.

## Data Availability

Not applicable.
